# N-3 PUFA Deficiency Aggravates Streptozotocin-Induced Pancreatic Injury in Mice but Dietary Supplementation with DHA/EPA Protects the Pancreas via Suppressing Inflammation, Oxidative Stress and Apoptosis

**DOI:** 10.3390/md21010039

**Published:** 2023-01-01

**Authors:** Hong-Yu Zou, Hui-Juan Zhang, Ying-Cai Zhao, Xiao-Yue Li, Yu-Ming Wang, Tian-Tian Zhang, Chang-Hu Xue

**Affiliations:** 1 College of Food Science and Engineering, Ocean University of China, No.1299 Sansha Road, Qingdao 266404, China; 2 Laboratory for Marine Drugs and Bioproducts, Pilot National Laboratory for Marine Science and Technology (Qingdao), Qingdao 266237, China

**Keywords:** streptozotocin, polyunsaturated fatty acid, pancreas injury, oxidative stress, inflammation, apoptosis, mouse

## Abstract

It has been reported that dietary n-3 polyunsaturated fatty acids (n-3 PUFAs) exert therapeutic potential for the preservation of functional β-cell mass. However, the effect of dietary n-3 PUFA deficiency on pancreatic injury and whether the supplementation of n-3 PUFA could prevent the development of pancreatic injury are still not clear. In the present study, an n-3 PUFA deficiency mouse model was established by feeding them with n-3 PUFA deficiency diets for 30 days. Results showed that n-3 PUFA deficiency aggravated streptozotocin (STZ)-induced pancreas injury by reducing the insulin level by 18.21% and the HOMA β-cell indices by 31.13% and the area of islet by 52.58% compared with the STZ group. Moreover, pre-intervention with DHA and EPA for 15 days could alleviate STZ-induced pancreas damage by increasing the insulin level by 55.26% and 44.33%, the HOMA β-cell indices by 118.81% and 157.26% and reversed the area of islet by 196.75% and 205.57% compared to the n-3 Def group, and the effects were significant compared to γ-linolenic acid (GLA) and alpha-linolenic acid (ALA) treatment. The possible underlying mechanisms indicated that EPA and DHA significantly reduced the ration of n-6 PUFA to n-3 PUFA and then inhibited oxidative stress, inflammation and islet β-cell apoptosis levels in pancreas tissue. The results might provide insights into the prevention and alleviation of pancreas injury by dietary intervention with PUFAs and provide a theoretical basis for their application in functional foods.

## 1. Introduction

Type 1 diabetes mellitus (T1DM) is an autoimmune disease characterized by extreme insulin deficiency and resultant hyperglycemia resulting from pancreatic β-cell apoptosis [1]. With the rapid development of society, the growing incidence of T1DM presents a significant global public-health problem and a substantial global economic burden [1,2]. The statistical data of the International Diabetes Federation (IDF) show that more than 450 million people suffered from diabetes worldwide in 2017 and this number is predicted to increase to 693 million by 2045 [3]. Moreover, it has been estimated that T1DM accounts for approximately 5–10% of the total prevalence of diabetes throughout the world [4]. It is evidenced that protecting pancreatic β cells against damage or death is regarded as a novel therapeutic target for T1DM [5,6]. Therefore, it is of great necessity to prevent and control pancreas injury. 

It is evidenced that dietary n-3 polyunsaturated fatty acids (n-3 PUFAs) exert therapeutic potential for the preservation of functional β-cell mass [7]. N-3 PUFAs have protective effects on β-cell function via the normalization of insulin secretion in response to glucose in tunicamycin-treated islets [7]. Furthermore, it was observed that pre-intervention of EPA could significantly reduce the apoptosis compared to the tunicamycin-treated islets [7]. Another study also demonstrated that therapeutic intervention in nonobese diabetic (NOD) mice through nutritional supplementation or lentivirus-mediated expression of an n-3 fatty acid desaturase, m*fat-*1, normalized blood glucose and insulin levels, reduced inflammatory factor levels, such as IFN-γ, IL-17, IL-6 and TNF-α, prevented lymphocyte infiltration into regenerated islets and sharply elevated the expression of the β-cell markers pancreatic and duodenal homeobox 1 (*Pdx1*) and paired box 4 (*Pax4*) [8]. It has been reported that alpha linolenic acids (ALAs) could inhibit streptozotocin-induced pancreas injury, reduce serum insulin and glucose levels and restore δ6 desaturase activity and mRNA expression levels [9]. Bi et al. reported that n-3 PUFAs could both stall autoimmunity and fully restore pancreatic β-cell function [9]. Our previous study showed that deficiency of n-3 PUFA could aggravate inflammatory status and oxidative stress in the brain of mice [10]. Mounting evidence implies that reducing inflammation and oxidative stress could protect against the development of pancreatic injury and insulitis in rats/mice with T1DM [11,12]. However, the effect of dietary n-3 PUFA deficiency on pancreatic injury and whether the supplementation of n-3 PUFA could prevent the development of pancreatic injury are still not clear. 

The aim of the present study was to evaluate the effects of dietary n-3 PUFA levels on pancreatic injury. Therefore, an n-3 PUFA deficiency mouse model was established to investigate whether the deficiency of n-3 PUFA could aggravate STZ-induced pancreatic injury, and the preventive effects of dietary different types of n-3 PUFAs on pancreatic injury were also evaluated by pre-intervention of alpha-linolenic acid (ALA), eicosapentaenoic acid (EPA) and docosahexaenoic acid (DHA) for 15 days, in which gamma-linolenic acid (GLA), a kind of n-6 PUFA with anti-inflammatory activity, was used as a control. The pancreatic injury was induced by a single intraperitoneal injection of streptozotocin. The levels of fasting blood glucose and insulin, as well as histological changes in the pancreas, were determined. Moreover, the possible underlying mechanism was further clarified by determining the gene or protein expression levels of related indicators in oxidative stress, inflammation and apoptosis. Our study may be beneficial for further understanding the effects of dietary PUFAs on preventing pancreas injury and provide a theoretical basis for their application in functional foods.

## 2. Results and Discussion

### 2.1. Effects of Pre-Intervention with Different Types of PUFAs on Glucose Homeostasis and Islet β-Cell Function Homeostasis

**Figure 1:**
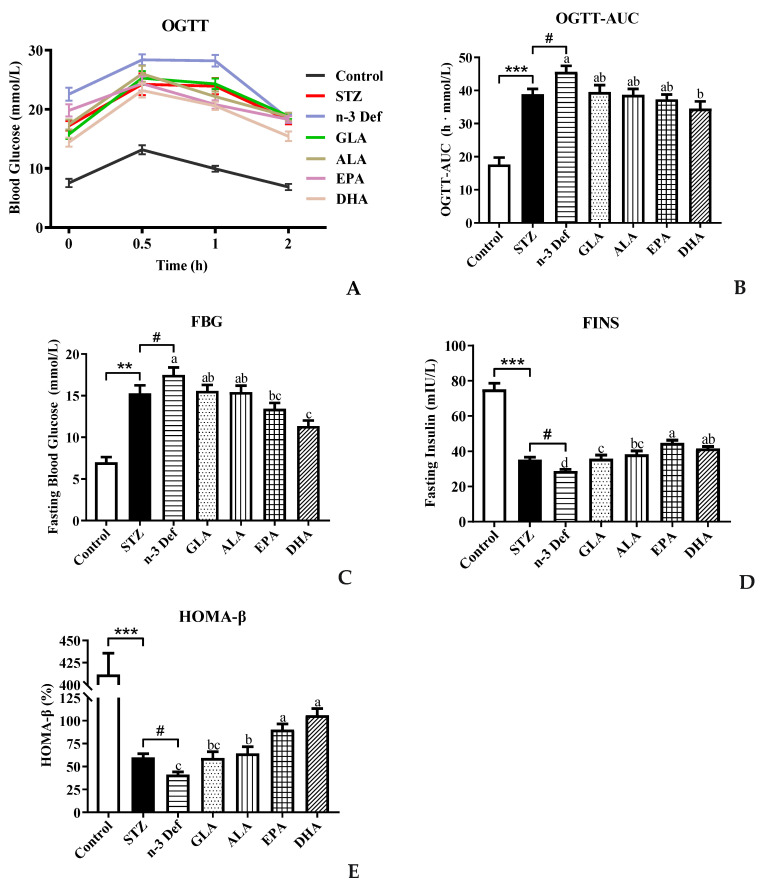
Effects of pre-intervention with different types of PUFAs on glucose homeostasis and islet β-cell function homeostasis. (**A**) Oral glucose-tolerance test and (**B**) area under the curve; (**C**) fasting blood glucose (FBG) levels after sacrifice; (**D**) fasting insulin (FINS) levels after sacrifice; (**E**) function index of β cells (HOMA-β), calculated by FBG and FINS. Results were presented as Mean ± SEM (*n* = 8) for each group. Significance analysis between Control and STZ, STZ and n-3 Def was performed using two-tailed Student’s *t*-test. ** *p* < 0.01, *** *p* < 0.001, compared to control group. # *p* < 0.05, compared to STZ group. Significance analysis among n-3 Def, GLA, ALA, EPA, DHA was performed using one-way ANOVA followed by Duncan’s multiple range test. Different letters indicated significant differences at *p* < 0.05 among pre-intervention groups.

To investigate the effect of different types of PUFA pre-intervention on glucose metabolism in normal mice, OGTT was performed before intraperitoneal injection of STZ. The result showed no significance among all groups (data not shown). As shown in Figure 1A,B, compared with the Control group, blood glucose levels at all indicated time points and the corresponding AUC were observed to be significantly (*p* < 0.001) high with STZ treatment. Notably, the AUC level in the n-3 Def group was significantly (*p* < 0.05) higher than that of the STZ group, indicating that the deficiency of n-3 PUFAs exacerbated the disorder of glucose metabolism. Compared with the n-3 Def group, following pre-intervention with GLA, ALA, EPA and DHA for 15 days, the AUC values were decreased by 13.29%, 15.17%, 18.17% and 24.32%, respectively. Interestingly, only the pre-intervention with DHA significantly decreased the AUC values (*p* < 0.05) compared to that in the n-3 Def group, and there was no significant difference among n-3 Def, GLA, ALA and EPA groups. 

Moreover, the fasting blood glucose level in the STZ group was significantly (*p* < 0.01) increased compared to that in the Control group (Figure 1C), implying that STZ treatment caused damage on the glucose homeostasis of mice. The fasting blood glucose level in the n-3 Def group was significantly (*p* < 0.05) higher than that in the STZ group, which suggested that deficiency of n-3 PUFAs exacerbated STZ-induced disorder of glucose metabolism. Compared to the n-3 Def group, a significant decrease (*p* < 0.05) in the fasting blood glucose level was found in the EPA and DHA groups, which was decreased by 23.2% and 35.1%, respectively. These results suggested that pre-intervention with DHA could effectively reduce glucose tolerance and maintain glucose homeostasis. The fasting blood insulin level is the direct reflection of insulin secretory function, which is the first indicator of islet β-cell function. The index of HOMA-β is usually used to evaluate the β-cell function [13]. As observed in Figure 1D, the fasting blood insulin level in the STZ group was significantly (*p* < 0.001) decreased in comparison with the Control group. Meanwhile, the fasting blood insulin level in the n-3 Def group was significantly (*p* < 0.05) higher than that in the STZ group, indicating that STZ treatment could destroy the insulin secretory function of mice and n-3 PUFA deficiency exacerbated this damage. Compared to the n-3 Def group, pre-intervention with GLA, ALA, EPA and DHA significantly (*p* < 0.05) increased fasting blood insulin levels by 24.25%, 32.88%, 55.26% and 44.33%, respectively. It has been reported that supplementation of EPA and DHA could significantly attenuate hyperglycemia and increase insulin levels in db/db mice, which was consistent with our results [14]. Wei et al. evaluated the direct impact of n-3 PUFAs on the functions and viability of pancreatic β cells by using isolated islets from *mfat-1* mice. They found that a cellular increase in n-3 PUFAs and reduction in n-6 PUFAs through transgenic expression of *mfat-1* enhanced glucose-, amino acid- and GLP-1-stimulated insulin secretion in isolated pancreatic islets, which was consistent with our result [15]. The index of HOMA-β was significantly (*p* < 0.001) decreased in the STZ group compared with the Control group, and n-3 PUFA deficiency further aggravated the decline in STZ-induced HOMA-β index (*p* < 0.05) (Figure 1E). Compared to the n-3 Def group, pre-intervention with GLA, ALA, EPA and DHA increased the index of HOMA-β by 43.94%, 55.95%, 118.81% and 157.26%, respectively, suggesting that islet β-cell function was improved with pre-intervention of different types of n-3 PUFAs and DHA exerted the best effect. Pinel et al. reported that intervention of n-3 PUFA could lead to an enhancement in insulin secretory activity and then protect islet β cells against streptozotocin-dependent injury, which was consistent with the present results [16]. These results underlined the possibility that pre-intervention of EPA and DHA might reduce hyperglycemia in mice with STZ-induced pancreas injury by improving pancreatic β-cell function and insulin secretion ability. All the above-mentioned results revealed that DHA and EPA were more effective than GLA and ALA in maintaining glucose homeostasis and islet β-cell function homeostasis. 

### 2.2. Effects of Pre-Intervention with Different Types of PUFAs on the Morphology of Pancreatic Tissue and mRNA Expressions of Genes Related to Pancreatic β-Cell Function

**Figure 2:**
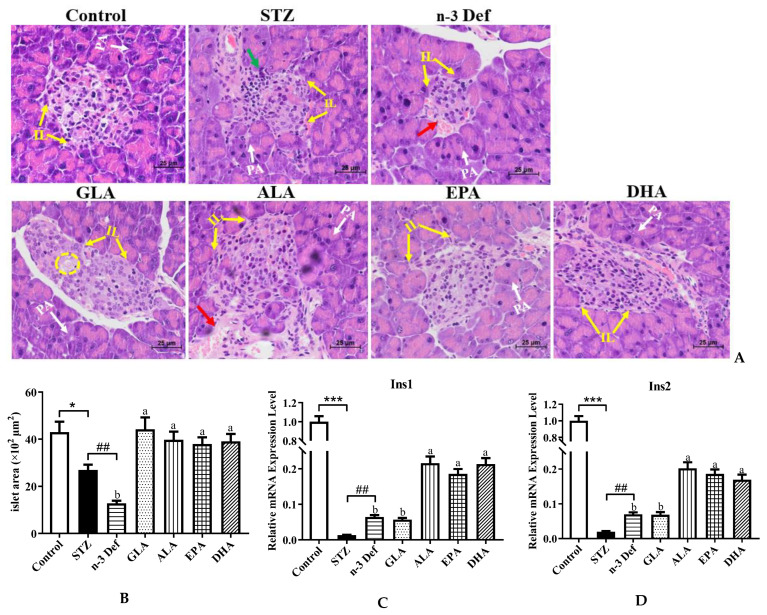
Effects of pre-intervention with different types of PUFAs on the morphology and function of pancreatic tissue. (**A**) Representative histopathological images of H&E staining of pancreas (400 × magnification). IL: islets of Langerhans; PA: pancreatic acini; red arrow: hemorrhage; green arrow: inflammatory infiltrate; yellow cycle: vacuolation. (**B**) Islet area of islet. The mRNA expression levels of Ins1 (**C**) and Ins2 (**D**) in pancreas. Results were presented as Mean ± SEM (*n* = 8) for each group. Significance analysis between Control and STZ, STZ and n-3 Def was performed using two-tailed Student’s *t*-test. * *p* < 0.05, *** *p* < 0.001, compared to Control group., ## *p* < 0.01, compared to STZ group. Significance analysis among n-3 Def, GLA, ALA, EPA, DHA was performed using one-way ANOVA followed by Duncan’s multiple range test. Different letters indicated significant differences at *p* < 0.05 among pre-intervention groups.

As portrayed in Figure 2A,B, the histopathological results of the Control group demonstrated complete pancreatic structure, normal pancreatic acini and central normal Langerhans islets with uniform arrangement of numerous pancreatic β-cells, while mild inflammatory cell infiltration and an obvious (*p* < 0.05) decrease by 37.38% area of Langerhans islets were observed in the STZ group compared with the Control group. The results confirmed selective cytotoxicity of STZ to the pancreas, which was in accordance with a previous study [17]. More severely, the n-3 Def group exhibited a dramatic (*p* < 0.01) decrease by 52.58% in area compared to the STZ group and obvious distortion of Langerhans islets, reduction in pancreatic β cells, moderate edema and a little hemorrhage. An improvement to different extents in the histological architecture and integrity of islets was found with pre-intervention of four kinds of PUFAs. Specifically, the area of Langerhans islets was significantly (*p* < 0.05) enlarged following pre-intervention of four kinds of PUFAs compared to the n-3 Def group, while there was no significant difference found among four kinds of PUFA groups. Pre-intervention of DHA seemed to be the most effective way to protect islets from damage, since there was a slight vacuolation and edema in islets in the GLA group, a little hemorrhage in the ALA group and less pancreatic β cells in the EPA group compared to the DHA group. The morphology results suggested that STZ injection contributed to the injury of pancreatic β cell and n-3 PUFA deficiency aggravated this condition. Importantly, pre-intervention of DHA exhibited an excellent effect on restoring pancreatic β cells.

When islet β cells were stimulated by glucose, Ins1 and Ins2 responsible for folding, processing and secretion of insulin were activated [18]. To assess the function of pancreas and insulin secretion ability of islet β cells, we determined the mRNA expressions of Ins1 and Ins2. The mRNA expression levels of Ins1 and Ins2 were dramatically (*p* < 0.001) reduced after STZ administration compared to those of the Control group (Figure 2B,C). Unexpectedly, n-3 PUFA deficiency displayed a significant increase in the expressions of Ins1 and Ins2, which needs further study. Following pre-intervention of ALA, EPA and DHA, the expression levels of Ins1 and Ins2 were significantly (*p* < 0.05) upregulated in comparison to those in the n-3 Def group. Shehata et al. suggested that the upregulation of Ins genes could improve pancreatic function and ameliorate STZ-induced diabetes, which was in line with our findings [19]. These results suggested that pre-intervention of DHA could significantly improve pancreatic function and restore the structure of the pancreas among PUFA-treated groups.

### 2.3. Effects of Pre-Intervention with Different Types of PUFAs on Oxidative Stress Levels and Relative mRNA Expression of Major Antioxidant Enzymes in Pancreatic Tissue

**Figure 3:**
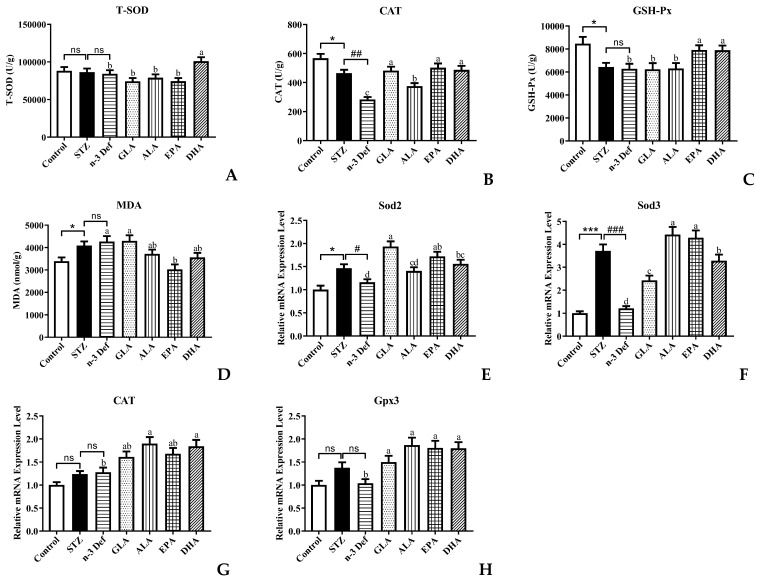
Effects of pre-intervention with different types of PUFAs on oxidative stress level and relative mRNA expression of major antioxidant enzymes in pancreatic tissue. The antioxidant enzyme activity of T-SOD (**A**), CAT (**B**), GSH-Px (**C**) in pancreas. The lipid peroxide contents of MDA (**D**) in pancreas. The mRNA expression levels of Sod2 (**E**), Sod3 (**F**), CAT (**G**), Gpx3 (**H**) in pancreas. Results were presented as Mean ± SEM (*n* = 8) for each group. Significance analysis between Control and STZ, STZ and n-3 Def was performed using two-tailed Student’s *t*-test. * *p* < 0.05, *** *p* < 0.001, compared to Control group. # *p* < 0.05, ## *p* < 0.01, ### *p* < 0.001, compared to STZ group. Significance analysis among n-3 Def, GLA, ALA, EPA, DHA was performed using one-way ANOVA followed by Duncan’s multiple range test. Different letters indicated significant differences at *p* < 0.05 among pre-intervention groups. ns: no significance.

Oxidative stress is one of the most important mechanisms in the process of pancreatic injury, dysfunction and apoptosis [20]. Pancreatic islet β cells are protected against reactive oxygen species (ROS) by endogenous antioxidant enzymes, including T-SOD, CAT and GSH-Px [21]. MDA is an indicator of lipid peroxidation. Decreased activity of antioxidant enzymes and accumulation of MDA result in damage to pancreatic islet β cells. Thus, to evaluate the oxidative stress levels in pancreatic islet β cells, the activity and mRNA expressions of the above-mentioned antioxidant enzymes as well as MDA level in pancreas were measured (Figure 3). After STZ administration, the activity of CAT and GSH-Px was significantly (*p* < 0.05) decreased and the content of MDA was significantly (*p* < 0.05) increased compared to the Control group, which suggested that STZ caused the oxidative damage to the pancreas. It has been reported that the levels of hydrogen peroxide, superoxide and lipid peroxides were significantly increased and the antioxidant enzyme activity was significantly decreased in the rat pancreas after intraperitoneal injection of STZ [22]. Moreover, deficiency of n-3 PUFA accentuated the decrease in CAT enzyme activity. With pre-intervention of EPA and DHA, the activity of CAT and GSH-Px was significantly (*p* < 0.05) increased and only DHA could significantly increase T-SOD activity compared with the n-3 Def group, which suggested that DHA and EPA possessed the scavenging capacity of free radicals [23], whereas GLA and ALA showed no significant effects on the elevation of T-SOD and GSH-Px activity and the reduction in MDA level. 

Thereafter, we examined the mRNA expression levels of related genes of antioxidant enzymes. The expression levels of Sod2 and Sod3 in the STZ group were significantly elevated compared with those in the Control group (Figure 3E,F). Notably, compared to the STZ group, deficiency of n-3 PUFA contributed to a significant decrease in the expression levels of Sod2 and Sod3. Of note, pre-intervention with GLA, EPA and DHA significantly increased the mRNA expression of Sod2 by 67.03%, 48.71% and 34.59%, respectively, compared with the n-3 Def group. Meanwhile, the mRNA expression of Sod3 was increased by 100%, 264.71%, 252.94% and 170.59%, respectively, after pre-intervention with GLA, ALA, EPA and DHA. There was no significant difference in the mRNA expressions of CAT and Gpx3 between the Control group and STZ group or the STZ group and n-3 Def group (Figure 3G,H). When pre-intervention with ALA and DHA was carried out, the CAT expression level was significantly (*p* < 0.05) upregulated by 48.73% and 44.20%, respectively, compared to that of the n-3 Def group. Nevertheless, no significant difference was found among the n-3 Def, ALA and EPA groups. Moreover, the Gpx3 mRNA expression level after pre-intervention of GLA, ALA, EPA and DHA was significantly upregulated by 44.26%, 79.97%, 74.02% and 73.29%, respectively, compared to that of the n-3 Def group. Corresponding with our results, previous work demonstrated that oxidative stress status in the pancreas was attenuated by downregulating activity and expression levels of antioxidative enzymes, including SOD, CAT and GSH-Px [24].

### 2.4. Effects of Pre-Intervention with Different Types of PUFAs on Inflammation 

**Figure 4:**
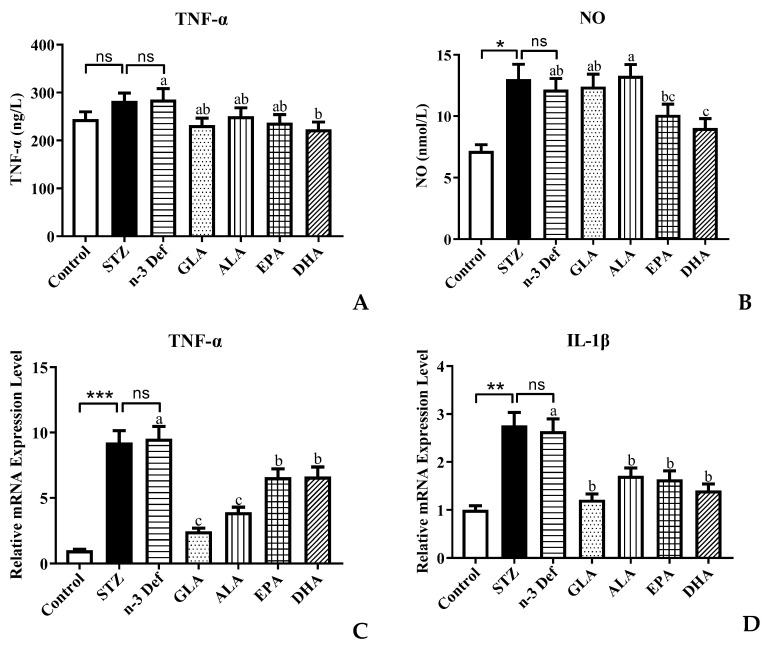
Effects of pre-intervention with different types of PUFAs on TNF-α/NO in serum and relative mRNA expression of TNF-α/ IL-1β in pancreatic tissue. The inflammatory factor levels of TNF-α (**A**) and NO (**B**) in serum. The mRNA expression levels of TNF-α (**C**) and IL-1β (**D**) in serum. Results were presented as Mean ± SEM (*n* = 8) for each group. Significance analysis between Control and STZ, STZ and n-3 Def was performed using two-tailed Student’s *t*-test. * *p* < 0.05, ** *p* < 0.01, *** *p* < 0.001, compared to Control group. Significance analysis among n-3 Def, GLA, ALA, EPA, DHA was performed using one-way ANOVA followed by Duncan’s multiple range test. Different letters indicated significant differences at *p* < 0.05 among pre-intervention groups. ns: no significance.

An increasing amount of evidence shows that there is a strong relationship between the inflammatory processes and β-cell dysfunction and apoptosis [25]. Activated macrophages produce the appearance of proinflammatory cytokines, which are associated with the upregulation of inflammation, promoting pancreatic β-cell apoptosis [26]. Inflammatory cytokines, such as TNF-α, IL-Iβ and NO, have been established to play a significant role in the pancreatic β-cell cytotoxic reactions and the insulitis that occurs in type 1 autoimmune diabetes [12]. STZ is a toxin, which leads to oxidative stress, inflammation and apoptosis of β cells in the pancreas mimicking autoimmune diabetes [26]. Over production of NO caused by immunological and inflammatory stimulation is considered as an important molecular mechanism leading to apoptosis of pancreatic β cells. Thus, the levels of NO and TNF-α were measured and results are shown in Figure 4. Following pre-intervention with different types of PUFA, the levels of TNF-α and NO in serum were significantly (*p* < 0.05) reduced only for pre-intervention of DHA compared to the n-3 Def group (Figure 4A,B). A previous study by Ganugula et al. reported that treatment with DHA could reduce TNF-α and NO levels in peritoneal macrophage obtained from STZ-induced diabetic mice to attenuate the inflammatory state, which was consistent with our findings [27]. However, dietary pre-intervention of GLA, ALA and EPA exhibited no significant effect on the TNF-α and NO level. This indicates that DHA could improve the pancreatic β-cell dysfunction by regulating the level of inflammatory cytokines. 

To further verify the regulating effect of DHA on inflammatory processes, the expressions of genes related to inflammation were examined. In comparison to the Control group, the mRNA expression level of TNF-α and IL-1β in the STZ group was significantly (*p* < 0.001, *p* < 0.01) increased. This confirms that streptozotocin, indeed, accentuated the inflammatory processes and subsequently led to pancreatic β-cell apoptosis, while there was no significant difference between the STZ and n-3 Def group. The expression levels of TNF-α in all groups were significantly (*p* < 0.05) reduced by 18.74%, 12.10%, 16.77% and 21.78%, respectively, compared with the n-3 Def group. This result was inconsistent with the TNF-α level in serum, which needs further study in the future. When there was pre-intervention with GLA, ALA, EPA and DHA, the expression level of IL-1β was significantly (*p* < 0.05) reduced by 54.12%, 35.35%, 38.08% and 46.83%, respectively. Corresponding with our results, a previous study showed that supplementation of n-3 PUFA inhibited inflammatory reaction in traumatic brain-injury-induced microglial activation by reducing the expression of inflammatory factors, including TNF-α, IL-1β, IL-6 and IFN-γ, in lesioned cortices [28]. 

Taken together, pre-intervention of DHA seems a more effective way to attenuate inflammatory processes and further prevent pancreatic β-cell apoptosis. It has been reported that resolvin D1, the anti-inflammatory metabolite of DHA, could attenuate the severity of STZ-induced T1DM by anti-inflammation, anti-oxidation and anti-apoptosis and by activating the *Pdx* gene that is needed for pancreatic β-cell proliferation [29]. We speculated that this might be the reason for the significant effect of DHA on inhibiting the inflammatory process.

### 2.5. Effects of Pre-Intervention with Different Types of PUFAs on the Expression of Apoptosis-Related Genes and Proteins in Pancreatic Tissue

**Figure 5:**
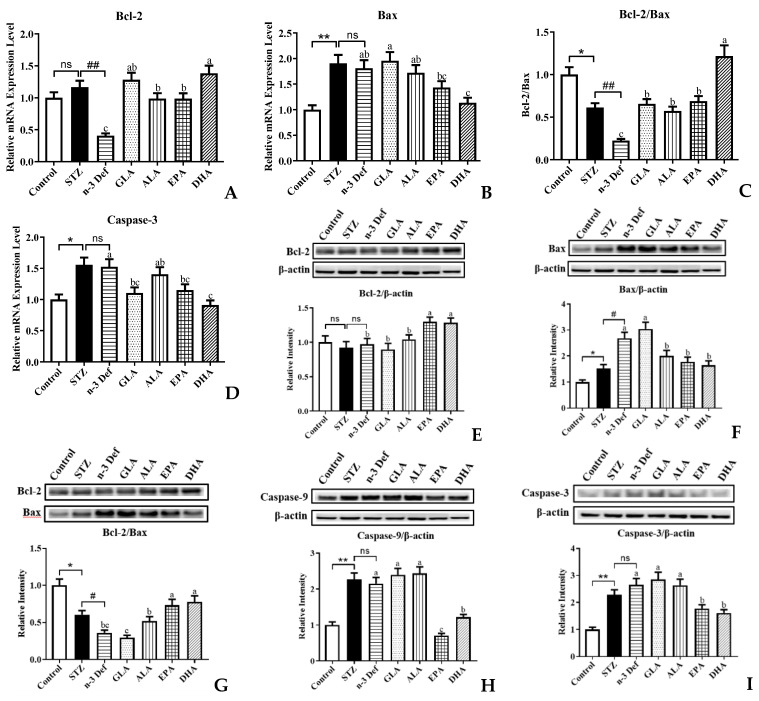
Effects of pre-intervention with different types of PUFAs on the expression of apoptosis-related genes and proteins in pancreatic tissue. The mRNA expression levels of mitochondrial apoptosis pathway, including Bcl-2 (**A**), Bax (**B**), Bcl-2/ Bax (**C**), Caspase-3 (**D**). The protein expression levels of mitochondrial apoptosis pathway, including Bcl-2 (**E**), Bax (**F**), Bcl-2/ Bax (**G**), Caspase-9 (**H**), Caspase-3 (**L**). Results were presented as Mean ± SEM (*n* = 8) for each group. Significance analysis between Control and STZ, STZ and n-3 Def was performed using two-tailed Student’s *t*-test. * *p* < 0.05, ** *p* < 0.01, compared to Control group. # *p* < 0.05, ## *p* < 0.01, compared to STZ group. Significance analysis among n-3 Def, GLA, ALA, EPA, DHA was performed using one-way ANOVA followed by Duncan’s multiple range test. Different letters indicated significant differences at *p* < 0.05 among pre-intervention groups. ns: no significance.

It is well established that the mitochondrial apoptosis pathway of pancreatic β cells is the intrinsic mechanism for pancreas injury, where the pro-apoptotic factors are considered as potential therapeutic targets [30]. To gain insight into the effects that different types of PUFAs inhibit β-cell apoptosis, we examined the expressions of genes and proteins involved in the mitochondrial apoptosis pathway (Figure 5). As revealed by quantitative real-time PCR analysis, deficiency of n-3 PUFA significantly (*p* < 0.01) inhibited the mRNA expression of anti-apoptotic gene Bcl-2 compared to the STZ group (Figure 5A). Pre-intervention of four kinds of PUFAs significantly (*p* < 0.05) attenuated these terrible changes, especially DHA, which exhibited outstanding effects among four pre-interventions. Figure 5B,D showed that the mRNA expressions of pro-apoptotic genes, Bax and Caspase-3, were significantly (*p* < 0.01, *p* < 0.05) upregulated followed by STZ administration, compared with the Control group. There were no significant differences in Bax and Caspase-3 gene expressions between the STZ and n-3 Def group. In terms of gene expression of Bax, only pre-intervention of DHA significantly (*p* < 0.05) reduced its expression levels compared to that in the n-3 Def group, whereas GLA, ALA and EPA showed no significance compared with the n-3 Def group. Compared to the n-3 Def group, pre-intervention of GLA, EPA and DHA significantly (*p* < 0.05) reduced the expression levels of Caspase-3 by 27.51%, 24.45% and 40.18%, respectively, while pre-intervention of ALA showed no significant effect. Administration with STZ significantly (*p* < 0.05) reduced the Bcl-2/Bax gene expression ratio and deficiency of n-3 PUFA significantly (*p* < 0.01) exacerbated the terrible changes (Figure 5C). Pre-intervention with four kinds of PUFAs significantly increased the Bcl-2/Bax gene expression ratio to different extents, particularly DHA, which exhibited the best effects on reversing against the reduction in Bcl-2/Bax gene expression ratio compared to the other three kinds of PUFAs. A previous study reported that berberine inhibited STZ-induced apoptosis in mouse pancreatic islets through downregulating Bax/Bcl-2 gene expression ratio, which was in accordance with our results [31]. 

Compared to the n-3 Def group, pre-intervention of EPA and DHA significantly increased the protein expression of Bcl-2, while GLA and ALA showed no significant effect (Figure 5E). Administration with STZ significantly increased pro-apoptotic protein expression levels, including Bax, Caspase-9 and Caspase-3 (Figure 5F,H,I). Moreover, n-3 PUFA deficiency significantly increased Bax protein expression levels compared with the STZ group (Figure 5F). The protein expression of Bax was significantly reduced by pre-intervention of ALA, EPA and DHA, while pre-intervention of GLA showed no significant effect (Figure 5F). The caspase cascade plays a significant role in pancreatic β-cell apoptosis via both extrinsic and intrinsic pathways [32]. Caspase-9 is an initiator of apoptosis, which is activated when it receives a signal and then responds to trigger effector caspase. Caspase-3 is the pivotal performer of cell apoptosis [32]. The results revealed that pre-intervention of EPA and DHA significantly (*p* < 0.05) downregulated the Caspase-9 and Caspase-3 protein expression levels. Notably, GLA and ALA had no significant effect on downregulating the protein expression of Caspase-9 and Caspase-3. Moreover, the Bcl-2/Bax protein expression ratio was significantly (*p* < 0.05) reduced by administration with STZ and n-3 PUFA deficiency significantly exacerbated this change (Figure 5G). Compared with the n-3 Def group, pre-intervention of ALA, EPA and DHA significantly (*p* < 0.05) increased the Bcl-2/Bax protein expression ratio by 43.52%, 102.58% and 115.51%, respectively. Similarly, syzygium jambos extract attenuated pancreatic apoptosis by reducing pro-apoptosis Caspase-3 and increasing anti-apoptotic Bcl-2 proteins [33]. A study by Wang et al. also evaluated the effect of EPA on apoptosis in isolated islets from mice and observed less apoptosis in the EPA-pretreated islets than in the tunicamycin-treated islets, including the decrease in Bax and cleaved caspase-3 and an increase in Bcl-2, which was also consistent with our results [7]. These results were in agreement with the result from quantitative real-time PCR analysis, which confirmed that n-3 PUFA deficiency could exacerbate STZ-induced pancreatic β-cell apoptosis and dietary supplementation with DHA and EPA could alleviate terrible changes.

### 2.6. Effects of Pre-Intervention with Different Types of PUFAs on Main PUFA Composition and n-6/n-3 Ratio in Pancreatic Tissue

A previous study reported that dietary supplementation with low n-6/n-3 ratio could improve blood glucose homeostasis, reduce systematic inflammation and ameliorate the progress of diabetes [34]. Moreover, it has been established that endogenous low n-6/n-3 ratio in pancreatic levels can be more of a benefit to manage STZ-induced diabetes [35]. Herein, fatty acid compositional analysis was performed to assess the n-6/n-3 ratio in pancreatic tissue. As the mother substance of the pro-inflammatory eicosanoids, arachidonic acid is released from membrane phospholipids when activated by inflammation and then is metabolized to prostaglandins and leukotrienes, accelerating the process of inflammatory disorders [36]. As shown in Table 1, there was no significant difference in arachidonic acid (AA; 20:4 n-6) and linoleic acid (LA; 18:2 n-6) among Control, STZ and n-3 Def groups. With pre-intervention of four kinds of PUFAs, AA level in pancreas tissue was significantly (*p* < 0.05) reduced by 24.29%, 40.51%, 57.35% and 59.85%, respectively. No significant difference was found in linoleic acid (LA; 18:2 n-6) among n-3 Def, ALA, GLA, EPA and DHA groups. N-3 PUFA was significantly (*p* < 0.05) reduced in the n-3 Def group compared with the STZ group. Moreover, pre-intervention of ALA, EPA and DHA increased n-3 PUFA level in pancreas tissue by 233.17%, 253.37% and 349.45%, respectively. There was no significant difference in the ratio of n-6 PUFA to n-3 PUFA between the Control and STZ group, and the ratio of n-6 PUFA to n-3 PUFA was significantly (*p* < 0.01) increased in the n-3 Def group, in comparison to the STZ group. Following pre-intervention of ALA, EPA and DHA, the ratio of n-6 PUFA to n-3 PUFA was significantly (*p* < 0.01) reduced by 74.90%, 80.90% and 81.60%, respectively, while pre-intervention of GLA showed no similar significant effect. The ratio of n-6 PUFA to n-3 PUFA in pancreatic tissue is regarded as one of the most selective markers of pancreatic inflammation; the higher the ratio, the greater the proinflammatory conditions [23]. It has been reported that low inflammatory index n-6/n-3 can protect against STZ-induced pancreatic injury, which may be one of the reasons for the best effect of DHA on protecting the pancreas [35]. It has been suggested that many EPA and DHA derivatives are signaling molecules and less-harmful compounds than the corresponding n-6 metabolites [37]. Our results indicated that pre-intervention of DHA could protect mice from STZ-induced pancreatic β-cell damage by reducing the inflammatory index n-6/n-3 and AA levels in pancreas tissue.

## 3. Materials and Methods

### 3.1. Materials

DHA-rich fish oil (Cat no: QYC-20200202-03) and EPA-rich fish oil (Cat no: QYC-20200202-02) were purchased from BenheBiotechnology Co., Ltd. (Xian, China). Flaxseed oil rich in ALA (Cat no: 120102321) and GLA (Cat no: 120103122) was purchased from Inno Biotechnology Co., Ltd. (Dalian, China). Streptozotocin (STZ. Cas no: 18883-66-4) was purchased from Aladdin Biochemical Technology Co., Ltd. (Shanghai, China). The glucose assay kit (Cat no: 100000240) was obtained from BioSino Biotechnology and Science Co., Ltd. (Beijing, China). The assay kits for total superoxide dismutase (T-SOD. Cat no: A001-1-1, Hydroxylamine method), catalase (CAT. Cat no: A007-1-1, ammonium molybdate method), glutathione peroxidase (GSH-Px. Cat no: A005-1-2, 2-Nitrobenzoic acid method) and malondialdehyde (MDA. Cat no: A003-1-2, Thiobarbituric acid method) were obtained from Nanjing Jiancheng Bioengineering Institute (Nanjing, China). The tumor necrosis factor-α (TNF-α. Cat no: H052-1-2, indirect) and insulin (Cat no: CK-1-20533-M, indirect) ELISA kits were, respectively, provided by Nanjing Jiancheng Bioengineering Institute (Nanjing, China) and Calvin Biotechnology Co., Ltd. (Suzhou, China). The assay kit for Nitric Oxide (NO, Cat no: S0021) was obtained from Beyotime Biotech Inc. (Wuhan, China). The primary Radio immunoprecipitation Assay (RIPA. Cat no: P0013B) lysis buffer was from Beyotime Biotechnology Co., Ltd. (Shanghai, China). The primary antibodies against β actin (Cat no: 3700T), B-cell lymphoma2 (Bcl-2. Cat no: 10571S), Bcl2-associated X protein (Bax. Cat no: 89477S), caspase-9 (Cat no: 9508T), caspase-3 (Cat no: 9668T) as well as the secondary antibodies (Cat no: 91196S) were purchased from Cell Signaling Technology (Beverly, MA, USA). The bicinchoninic acid (BCA. Cat no: P0010S) kit was purchased from Beyotime Biotech Inc. (Shanghai, China). The Trizol reagent (Cat no: 15596-026) was provided by Invitrogen (Carlsbad, CA, USA) and random primer was from Thermo Fisher Scientific, Inc. (Pittsburgh, PA, USA).

### 3.2. Animals and Treatments 

All animal experimental protocols complied with the Ethical Committee of Experimental Animal Care at the College of Food Science and Engineering, Ocean University of China (Qingdao, China) (Approval No. SPXY2019016). In total, 56 Male C57BL/6J mice (6–8 weeks) purchased from the Vital River Laboratory Animal Technology Co., Ltd. (Beijing, China) were kept in an environment with a constant temperature of 23 °C and relative humidity of 50–65% under a 12h/12h light/dark cycle. All mice were singularly caged. Furthermore, mice were provided with food in equivalent quantity and free access to water. 

All animals were provided with a commercial diet for one week. After one-week acclimatization, mice were randomly divided into 7 groups (*n* = 8): normal Control group (Control), STZ-injected Control group (STZ), n-3 PUFA-deficient group (n-3 Def), GLA pre-intervention group (GLA), ALA pre-intervention group (ALA), EPA pre-intervention group (EPA) and DHA pre-intervention group (DHA). Control and STZ groups were continuously fed with basic diet containing 0.31% (*w*/*w*) ALA for 30 days. N-3 Def group was continuously fed with n-3 PUFA-deficient diet for 30 days to reduce the n-3 PUFA level in mice. GLA, ALA, EPA and DHA groups were fed with a basic diet containing 0.31% (*w*/*w*) ALA for 15 days and then provided with a diet containing corresponding 1% (*w*/*w*) PUFA for 15 days. In addition, diet intakes were measured every day and body weights of animals were measured every two days during the experiment. On the 31st day, all mice received a single intraperitoneal injection of 150 mg/kg STZ dissolved in 0.1 M citrate buffer, pH 4.5 and mice in the normal Control group were injected with only the buffer solution, after 12 h fasting. Forty-eight hours after the injection, all mice were fasted for 12 h (free access to water) and then an oral glucose tolerance test (OGTT) was conducted. Two hours after OGTT test, all mice were euthanized with CO_2_ [38] and the tissue of pancreas was collected and dissected carefully. Then, part of pancreas tissue was fixed with 4% buffered paraformaldehyde for histological study, and the remaining was quickly frozen with liquid nitrogen and stored at −80 °C for further analysis. After the blood sample was kept at 4 °C for 30 min, the serum sample was obtained via centrifugation at 3500 rpm at 4 °C for 15 min. The serum was immediately stored at −80 °C until further analysis. 

### 3.3. Oral Glucose Tolerance Test 

An OGTT test was carried out after forty-eight hours of STZ injection as described previously [39]. After a 12 h overnight fast, all mice received oral treatment of glucose (2 g/kg body weight) and blood samples were collected from the tail vein at 0, 0.5, 1 and 2 h. The serum sample was obtained via centrifugation at 3500 rpm at 4 °C for 15 min and then the glucose assay kits were used to assay the serum glucose concentration. Additionally, the areas under the curve (AUC) of glucose were calculated.

### 3.4. Serum Biochemical Tests 

The levels of glucose and NO in the serum samples were assayed with corresponding enzymatic reagent kits. The concentrations of insulin and TNF-α were determined by corresponding ELISA reagent kits. All measurements were conducted in accordance with the manufacturer’s protocols. To assess the function of islet β cells in insulin secretion, the HOMA β-cell indices were calculated as 20 × fasting serum insulin (mIU/L)/fasting serum glucose (mmol/L) − 3.5 [40]. 

### 3.5. Indicators of Oxidative Stress in Pancreas

The levels of malondialdehyde (MDA) and antioxidant enzymes activity of total super oxide dismutase (T-SOD), glutathione peroxidase (GSH-Px) and catalase (CAT) were, respectively, measured in the pancreatic tissue homogenates using corresponding kits. All measurements were conducted according to the manufacturer’s instructions.

### 3.6. Histological Analysis

The pancreas tissue was fixed with 4% buffered paraformaldehyde, dehydrated using the graded ethanol series, embedded in paraffin and cut into 4–5 µm-thick tissue sections. The specimens were stained with hematoxylin and eosin (H&E) and evaluated histologically to determine the degree of pancreatic injury under a light microscope (Olympus BX41, Olympus Optical, Tokyo, Japan).

### 3.7. RNA Isolation and Real-Time PCR Analysis

Real-time polymerase chain reaction (RT-PCR) was used to measure the mRNA levels of associated genes. As previously described [41], RNA extraction and quantitative real-time PCR were carried out. Total RNA was extracted from the pancreas tissue using the Trizol reagent. Random primers and Moloney Murine Leukemia Virus Reverse Transcriptase were used to reverse-transcribe RNA into cDNA. Target Genes were amplified using the SYBR Green I Master Mix and 0.3 μM of both forward and reverse primers in the PCR detection system (Bio-Rad, Hercules, CA, USA). The expression levels of target genes were represented as normalization to the levels of β actin using the 2^−ΔΔCT^ method [42]. The primer sequences are shown in Table S1 in the Supporting Materials.

### 3.8. Western Blotting Analysis

Western blotting analysis was carried out in accordance with the procedures previously described [43]. Briefly, total proteins of pancreas were extracted using RIPA lysis buffer mixed with phenylmethylsulfonyl fluoride and phosphatase inhibitor. The BCA protein assay kit was used to detect protein concentrations. The protein (40 μg) was separated with 10% sodium dodecyl sulfate–polyacrylamide gel electrophoresis (SDS-PAGE) and then electroblotted to polyvinylidene fluoride membranes. All membranes were blocked in 5% BSA followed by washing, and subsequently they were incubated with primary antibodies of Bcl-2 (1:1000), Bax (1:1000), Caspase-9 (1:1000), Caspase-3 (1:1000) and β actin overnight at 4 ℃. After that, corresponding secondary antibodies were incubated with them for 1 h at room temperature and bands were detected using enhanced chemiluminescence under a UVP Auto Chemi Image system (UVP Inc., Upland, CA, USA). The target protein expression levels were quantified by Image J software (version 1.410) and normalized to the levels of β actin.

### 3.9. Fatty Acid Composition of Pancreas

Total lipid extraction of the pancreas was performed with chloroform and methanol, according to the general method of Bligh/Dyer [44]. In brief, following a water bath at 40 °C for 30 min, total pancreas lipids were extracted three times using a chloroform/methanol (1:1, *v*/*v*) solution. The chloroform layer was then obtained by centrifuging at 8000 rpm for 3 min at 4 °C. The mixtures of three repetitions of the chloroform layer are the pancreas lipid extraction. Pancreas fatty acid composition was analyzed by gas chromatography (GC). To perform a qualitative analysis of fatty acids, a mixture of 37 kinds of fatty acid methyl esters was used. The amount of relative fatty acid in pancreas was measured using an internal standard, TAG C15:0 (NU-CHEK Inc., Elysian, MN, USA). Briefly, 100 μL of the pancreas lipid extraction was added into a tube containing 30 μg TAG C15:0 as the internal standard and then the liquid was concentrated in vacuo. Then, 2 mL hydrochloric acid–methanol (1:5, *v*/*v*) was added into the tube filled with nitrogen (N2) and methylation was performed at 90 °C for 3 h with occasional shaking. After being cooled down to room temperature, 1.5 mL n-hexane was mixed and then hexane layer was removed and immediately injected into the GC. The analysis was conducted using a capillary column Supelcowax (30 mm × 0.32 mm I.D and 0.25 μm film thickness; Sigma-Aldrich Inc, St Louis, MO, USA). The nitrogen was used as carrier gas with a flow rate of 1.0 mL min 1. The temperature of the injector and detector was 240 °C and 260 °C, respectively. The column oven’s temperature was heated from 170 °C to 240 °C at a rate of 3 °C per min and it was kept at 240 °C for 15 min. The normalization method of the peak area was used to measure the relative content of each peak.

### 3.10. Statistical Analysis

Results were presented as the mean ± standard error of the mean (SEM) values. All data analysis was performed in SPSS 22.0 software. Statistical differences between Control group and STZ group, STZ and n-3 Def were performed using two-tailed Student’s *t*-test. * *p* < 0.05, ** *p* < 0.01, *** *p* < 0.001, compared to Control group. # *p* < 0.05, ## *p* < 0.01, ### *p* < 0.001, compared to STZ group. One-way analysis of variance (ANOVA) followed by Duncan’ test was performed for determining statistical differences among n-3 Def, GLA, ALA, EPA and DHA groups. The significant difference was obtained when *p* value < 0.05 and indicated by different letters.

## 4. Conclusions

In conclusion, the present study demonstrated that dietary n-3 PUFA deficiency aggravated STZ-induced pancreas injury presented by the decreased insulin secretion ability and the damaged pancreatic structure, which was accompanied by the increasing levels of oxidative markers, proinflammatory cytokines and apoptosis executors. Additionally, dietary supplementation of EPA and DHA for 15 days significantly prevented severe pancreas injury by improving glucose metabolic homeostasis and islet structural integrity, which exerted more significant effects than pre-intervention of GLA and ALA. Further mechanism research showed that the preventive effect of DHA and EPA on pancreatic injury was associated with the reducing levels of oxidative stress and the inhibition of inflammation and islet β-cell apoptosis. Furthermore, EPA and DHA significantly reduced the level of AA and ratio of n-6 PUFA to n-3 PUFA in pancreatic tissue. These findings may provide insights into the development of EPA and DHA as potential functional supplementations for prevention and alleviation of pancreas injury.

## Figures and Tables

**Table 1:** Main PUFA composition of pancreatic tissue.

Fatty Acids (%)	Control	STZ	N-3 Def	GLA	ALA	EPA	DHA
C18:2(LA)	7.81 ± 0.68	10.83 ± 1.25 ^ns^	8.09 ± 0.93 ^ns, bc^	6.16 ± 0.57 ^c^	10.01 ± 1.16 ^ab^	8.79 ± 1.01 ^bc^	12.37 ± 1.57 ^a^
C18:3(ALA)	0.23 ± 0.03	0.19 ± 0.02 ^ns^	ND	0.18 ± 0.02 ^b^	0.36 ± 0.04 ^a^	ND	0.12 ± 0.01 ^b^
C20:4(AA)	9.67 ± 1.23	9.91 ± 0.86 ^ns^	13.41 ± 1.24 ^ns, a^	10.16 ± 0.94 ^b^	7.98 ± 1.01 ^bc^	5.72 ± 0.72 ^c^	5.39 ± 0.68 ^c^
C20:5(EPA)	0.90 ± 0.11	1.79 ± 0.23 *	0.85 ± 0.11 ^#, c^	0.41 ± 0.05 ^c^	3.48 ± 0.44 ^a^	4.17 ± 0.53 ^a^	2.28 ± 0.29 ^b^
C22:6(DHA)	1.08 ± 0.14	1.41 ± 0.12 ^ns^	0.89 ± 0.08 ^#, bc^	0.66 ± 0.08 ^c^	1.95 ± 0.24 ^b^	1.96 ± 0.25 ^b^	5.40 ± 0.69 ^a^
n-6 PUFA	17.47 ± 1.41	20.73 ± 1.80 ^ns^	21.50 ± 1.74 ^ns, a^	16.32 ± 1.41 ^b^	17.99 ± 1.56 ^ab^	14.51 ± 1.26 ^b^	17.76 ± 1.33 ^ab^
n-3 PUFA	2.21 ± 0.17	2.89 ± 0.29 ^ns^	1.74 ± 0.14 ^#, c^	1.25 ± 0.11 ^c^	5.78 ± 0.50 ^b^	6.13 ± 0.50 ^b^	7.80 ± 0.59 ^a^
n-6/n-3 ratio	7.91 ± 0.46	6.12 ± 0.70 ^ns^	12.39 ± 0.93 ^##, a^	13.03 ± 0.90 ^a^	3.11 ± 0.20 ^b^	2.37 ± 0.22 ^b^	2.28 ± 0.20 ^b^

Note: ND, not detected. Results were presented as Mean ± SEM (*n* = 8) for each group. Significance analysis between Control and STZ, STZ and n-3 Def was performed using two-tailed Student’s *t*-test. * *p* < 0.05, compared to Control group. ^#^ *p* < 0.05, ^##^ *p* < 0.01, compared to STZ group. Significance analysis among n-3 Def, GLA, ALA, EPA, DHA was performed using one-way ANOVA followed by Duncan’s multiple range test. Different letters indicated significant differences at *p* < 0.05 among pre-intervention groups.

## Data Availability

Not applicable.

## Supplementary Materials

The following supporting information can be downloaded at: https://www.mdpi.com/article/10.3390/md21010039/s1, Figure S1: Effects of pre-intervention with different types of PUFA on body weights and food intakes in mice. (**A**) Mean food intake of mice during experiments. (**B**) Body weight of mice during experiments; Table S1: Ingredients of experimental diets of different kinds of PUFAs; Table S2: Primers used in this study.
