# Supplementary material for: N-3 PUFA Deficiency Aggravates Streptozotocin-Induced Pancreatic Injury in Mice but Dietary Supplementation with DHA/EPA Protects the Pancreas via Suppressing Inflammation, Oxidative Stress and Apoptosis
Source: Mar Drugs. 2023 Jan 1;21(1):39. doi: 10.3390/md21010039 (PMC9861647; doi:10.3390/md21010039)
Supplement: Supplementary file 1 [file marinedrugs-21-00039-s001.zip › marinedrugs-2099794-supplementary.pdf]

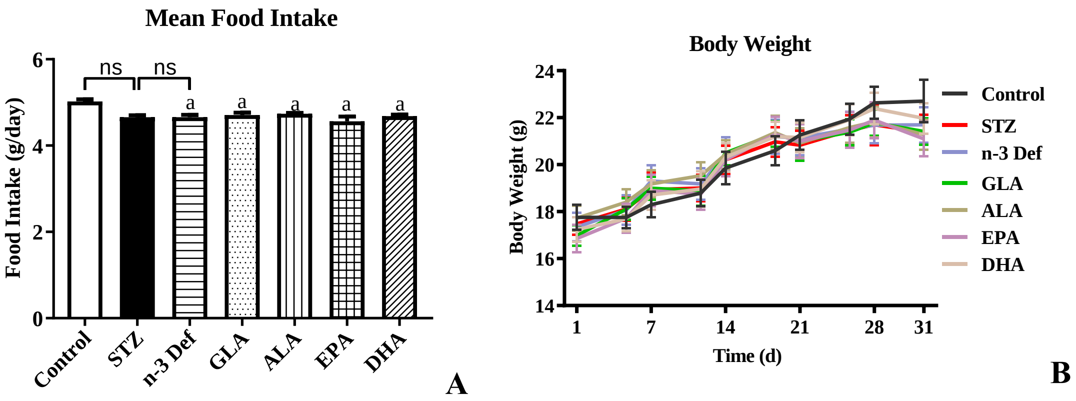

Figure S1. Effects of pre-intervention with different types of PUFA on body weights and food intakes in mice. Results were presented as Mean ± SEM (n=8) for each group. (A) Mean food intake of mice during experiments. (B) Body weight of mice during experiments.

Table S1. Ingredients of experimental diets of different kinds of PUFAs

Ingredients (g/kg)	CONTROL	STZ	N-3 Def	GLA	ALA	EPA	DHA
Potato starch	397.49	397.49	397.49	397.49	397.49	397.49	397.49
Casein	200	200	200	200	200	200	200
Sucrose	100	100	100	100	100	100	100
Maltodextrin	132	132	132	132	132	132	132
Cellulose	50	50	50	50	50	50	50
Mineral mix[a]	35	35	35	35	35	35	35
Vitamin mix[b]	10	10	10	10	10	10	10
L-methionine	3	3	3	3	3	3	3
Choline bitartrate	2.5	2.5	2.5	2.5	2.5	2.5	2.5
Hydrogenated coconut oil	52.56	52.56	56.7	45.37	43.2	45.39	45.33
Safflower oil	12.33	12.33	13.3	10.64	10.13	10.65	10.63
Linseed oil	5.11	5.11	--	--	16.67	--	--
Gamma-linolenic acid	--	--	--	13.99	--	--	--
DHA fish oil	--	--	--	--	--	13.96	--
EPA fish oil	--	--	--	--	--	--	14.04

[a] AIN-93 M mineral mix. b AIN-93 M vitamin mix. Note:“--”，not added

Table S2. Primers used in this study

Gene	Forward primer	Reverse primer
β-actin	GCAGATGTGGATCAGCAAGC	GTCAAAGAAAGGGTGTAAAACG
TNF-α	GGCAGGTCTACTTTGGAGTCATTGC	ACATTCGAGGCTCCAGTGAATTCGG
IL-1β	GTGTGTGACGTTCCCATTAGAC	GTCGTTGCTTGGTTCTCCTT
Bcl-2	CGGGAGAACAGGGTATGATA	CCACCGAACTCAAAGAAGG
Bax	TGCAGAGGATGATTGCTGAC	GATCAGCTCGGGCACTTTAG
Caspase-3	TGACTGGAAAGCCGAAACT	CTGGATGAACCACGACCC
Ins1	GAAGCGTGGCATTGTGGAT	TGGGCCTTAGTTGCAGTAGTTCT
Ins2	AGCCCTAAGTGATCCGCTACAA	CATGTTGAAACAATAACCTGGAAGA
Sod2	CACATTAACGCGCAGATCATG	CCAGAGCCTCGTGGTACTTCTC
Sod3	GCGTGTCGCCTATCTTCTCAA	AATGTCGCGTCTCTCTGAGG
CAT	GAACGAGGAGGAGAGGAAAC	TGAAATTCTTGACCGCTTTC
Gpx3	CCATTTGGCTTGGTCATTCTGGG	CACCTGGTCGAACATACTTGAGAC
